# CARM30: China annual rapeseed maps at 30 m spatial resolution from 2000 to 2022 using multi-source data

**DOI:** 10.1038/s41597-024-03188-1

**Published:** 2024-04-08

**Authors:** Wenbin Liu, Shu Li, Jianbin Tao, Xiangyu Liu, Guoying Yin, Yu Xia, Ting Wang, Hongyan Zhang

**Affiliations:** 1 Changjiang Institute of Survey Technical Research, MWR, Wuhan, Hubei 430011 China; 2 The State Key Laboratory of Information Engineering in Surveying, Mapping and Remote Sensing, Wuhan University, Wuhan, Hubei 430072 China; 3 The Key Laboratory for Geographical Process Analysis & Simulation of Hubei Province/School of Urban and Environmental Sciences, Central China Normal University, Wuhan, Hubei 430079 China; 4 Hubei Research Institute of Spatial Planning, Wuhan, Hubei 430064 China; 5 School of Computer Sciences, China University of Geosciences, Wuhan, Hubei 430074 China

**Keywords:** Solid Earth sciences, Agriculture

## Abstract

Rapeseed is a critical cash crop globally, and understanding its distribution can assist in refined agricultural management, ensuring a sustainable vegetable oil supply, and informing government decisions. China is the leading consumer and third-largest producer of rapeseed. However, there is a lack of widely available, long-term, and large-scale remotely sensed maps on rapeseed cultivation in China. Here this study utilizes multi-source data such as satellite images, GLDAS environmental variables, land cover maps, and terrain data to create the China annual rapeseed maps at 30 m spatial resolution from 2000 to 2022 (CARM30). Our product was validated using independent samples and showed average F1 scores of 0.869 and 0.971 for winter and spring rapeseed. The CARM30 has high spatial consistency with existing 10 m and 20 m rapeseed maps. Additionally, the CARM30-derived rapeseed planted area was significantly correlated with agricultural statistics (R^2^ = 0.65–0.86; p < 0.001). The obtained rapeseed distribution information can serve as a reference for stakeholders such as farmers, scientific communities, and decision-makers.

## Background & Summary

Rapeseed (*Brassica napus L*.) is a vital cash crop type, responsible for 13% of the world’s vegetable oil production^1–3^. It has been utilized for centuries as a source of cooking oils, animal feed, and protein meals^4,5^. In Europe, rapeseed production has experienced significant growth in recent decades due to the expansion of cropland and its potential for biofuels^6,7^. However, in many less-developed countries and regions, labor-intensive and smallholder-operated rapeseed cultivation is closely correlated with the rural population structure. The rural economy and agricultural plantation are subject to significant transformations due to the rapid marketization and urbanization processes^8–10^. Understanding the spatiotemporal dynamics of rapeseed can assist agricultural producers, scientific communities, and decision-makers in comprehending the potential of the vegetable-oil market, implementing refined agricultural management, and promoting the sustainable production of regional and national oilseeds.

Several publicly available remotely sensed land cover map products include specific rapeseed layers, such as the 30 m Cropland Data Layer produced by the United States Department of Agriculture and National Agricultural Statistics Service using moderate-resolution satellite imagery^11^, the 10 m European crop type map created by the European Commission using synthetic aperture radar (SAR) data and LUCAS Copernicus *in-situ* observations^12^, the 30 m Annual Crop Inventory product released by Canadian Agriculture and Agri-Food using both optical and SAR satellite images^13^, the 10 m annual Crop Map of England (CROME) from 2016 to 2020 produced by the Rural Payments Agency of the UK using Sentinel-1/2 imagery, and the 10 m RapeseedMap10 database across 33 countries in America and Europe published by Beijing Normal University, China using Sentinel-1/2 imagery^14^. These resources provide field-level information on rapeseed planting, benefiting government agencies, farmers, insurance companies, and other stakeholders and offering a promising future for the industry. However, despite these advancements, annual maps for national-scale rapeseed cultivation covering long period are currently limited due to a lack of ground survey samples, low availability of satellite imagery in cloudy areas, and regional differences in the phenology of rapeseed.

Rapeseed has a long history in China and occupies the largest planting area among oil crops, consistently providing over 13 million tons of oilseed to 1.4 billion people annually^15,16^. However, various factors such as rural labor being absorbed by urbanization, changes in vegetable oil consumption, and even an international oilseed trade deficit may impact enthusiasm for smallholder rapeseed planting^17–20^. To evaluate changes in rapeseed planting, Tao, *et al*.^18^ employed MODIS data to map the spatiotemporal dynamics of rapeseed cultivation in the middle reaches of the Yangtze River Valley of China in 2003 and 2015. However, the low resolution of 250 m MODIS data is not sufficient to adequately describe the fragmented landscape in southern China, let alone the intricate spatiotemporal dynamics. Liu and Zhang^21^ created rapeseed extent maps (REMs) at 10 m resolution in southern China using optical, SAR, and the Global Land Data Assimilation System (GLDAS) data, offering a detailed examination of rapeseed spatiotemporal patterns and revealing the process of field intensification. However, it is only capable of mapping winter rapeseed and ignores spring rapeseed produced in northern China. This gap was subsequently filled by the first nationwide 20 m annual rapeseed map for 2017 to 2021 generated by Zang, *et al*.^22^. Despite this, existing rapeseed maps are limited in terms of spatial accuracy, particularly in central and southern China, due to the scarcity of reliable ground samples and poor satellite observations caused by cloudy weather conditions^23,24^. Developing remote sensing mapping techniques that are more reliable and less dependent on ground samples is essential for accurate rapeseed monitoring.

Recent years have seen the proposal of various methods for mapping rapeseed, including (1) spectral-based methods^25,26^, (2) phenological-based methods^14,27,28^, (3) machine learning-derived methos^29^, and (4) collaborative mapping methods^21,22,24,30^. Rapeseed blooms are easily identifiable due to their distinctive bright yellow hue during flowering^31,32^. Many rapeseed mapping methods aim to improve the phenological or spectral separability of rapeseed from other contemporaneous crops, such as the normalized difference yellow index (NDYI)^27^ and the canola index (CI)^26^. However, due to the frequent cloud cover degrading the flowering image quality of rapeseed, these unsupervised classification approaches are challenging to deploy in southern China^24^. Machine learning classifiers can reduce reliance on flowering images for mapping rapeseed by inferring the characteristics distinguishing rapeseed from other crops using non-flowering variables^33^. However, the large-scale use of these classifiers can be hindered by the scarcity of well-represented training samples^34^. To properly address this issue, collaborative mapping strategies have been proposed to automatically generate training samples from existing datasets or phenological-based methods^33,35,36^. For instance, Zhang, *et al*.^24^ proposed a seamless and automated rapeseed mapping (SARM) method that fuses phenology and random forest (RF) classifiers and was applied in southern China^21^. Zang, *et al*.^22^ integrated a rule-based sample generation strategy and a one-class classifier to map the 20 m national-scale rapeseed maps from 2017 to 2021 in China. Despite some challenges, this collaborative mapping method, which combines unsupervised and supervised approaches, provides a feasible theoretical framework for creating high-precision and long-term rapeseed maps in China.

**Fig. 1:**
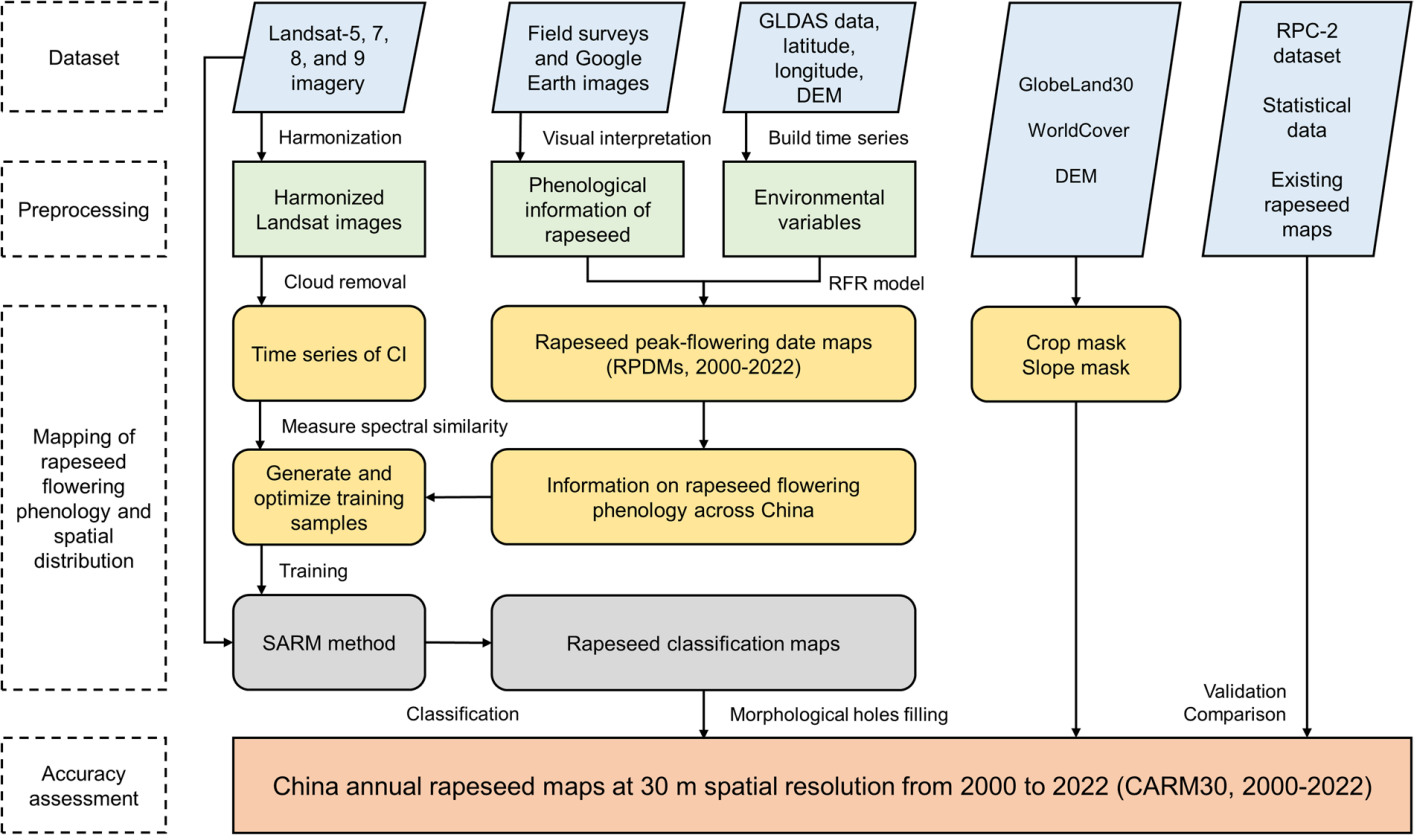
Workflow for mapping rapeseed in China.

In light of the aforementioned issues, we developed an automated method for creating a nation-scale, long-term rapeseed map at 30 m spatial resolution (CARM30) from 2000 to 2022, using multi-source data (Table S1). As illustrated in Fig. 1, our process comprises four components: (1) identifying rapeseed flowering time across China using field surveys, high-resolution satellite images, and GLDAS data; (2) generating and optimizing training samples for rapeseed mapping algorithm; (3) producing annual maps of rapeseed extent on Google Earth Engine (GEE); and (4) validating the accuracy of the CARM30 using independent ground samples, other existing rapeseed maps, and official agricultural statistics.

## Methods

### Study area

**Fig. 2:**
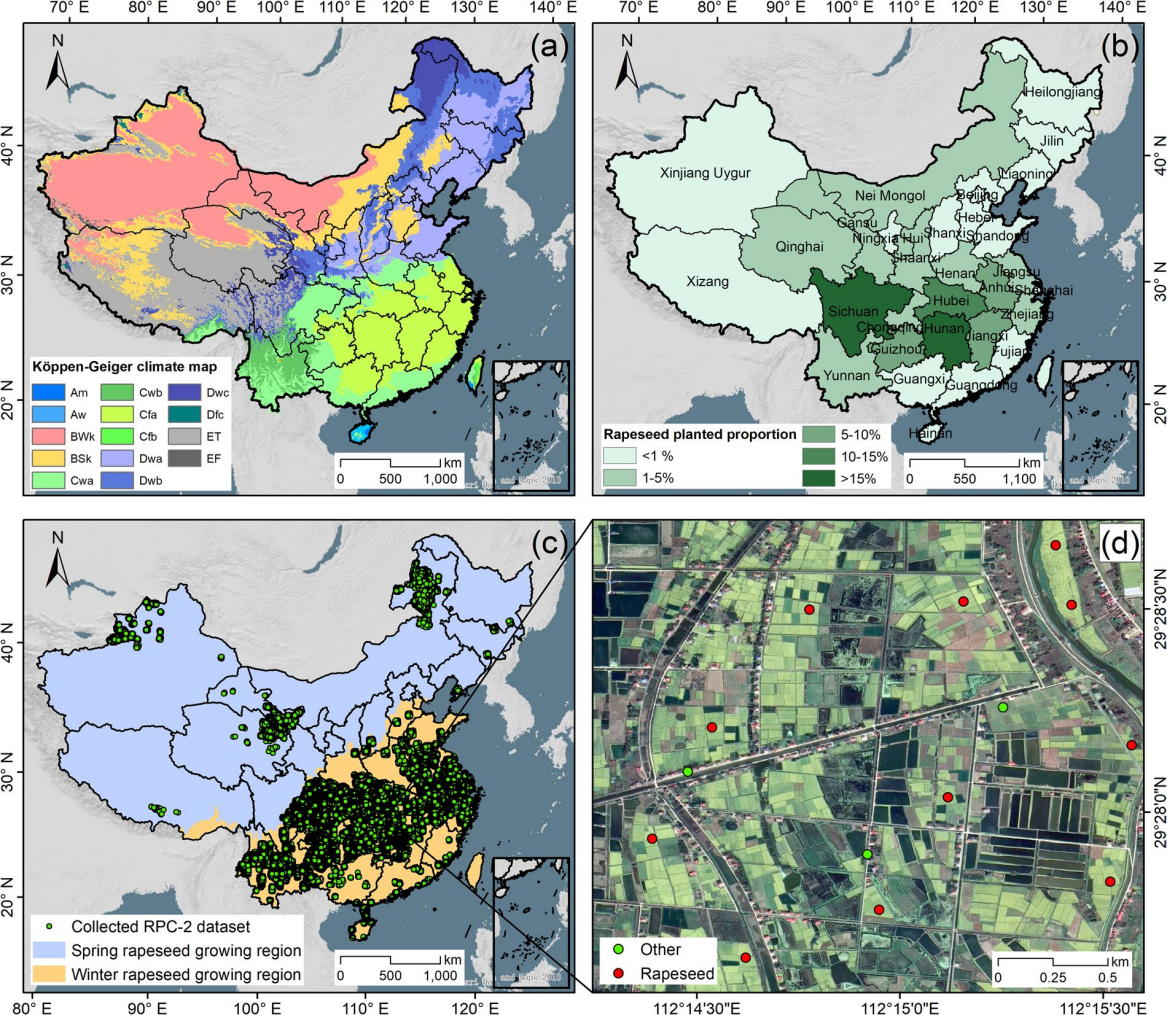
The study area. (**a**) Köppen-Geiger climate maps^76^, (**b**) proportion of rapeseed planted area at the provincial level, data from China Statistical Yearbook 2000–2021, (**c**) collected rapeseed point collection version 2 (RPC-2) dataset and the growing regions of spring and winter rapeseed, (**d**) a zoomed-in view of the RPC-2 dataset with overlay on the Google Earth © image.

China is the third-largest producer of rapeseed globally, following Canada and India^3,37^, with 24% of vegetable oil consumption coming from rapeseed^38^. Due to the diverse climate types (Fig. 2a), rapeseed in China is split into winter and spring variants depending on planting and vernalization timing^39,40^. Winter rapeseed is primarily found in southern China (Fig. 2b). It is typically planted from November until harvest before June in the following year, with a growing season of 210 to 230 days^37^. In contrast, spring rapeseed is planted in April and harvested before September after around 140 days of growth, mainly in Xinjiang, Qinghai, Gansu, and Nei Mongol^41^. To accurately map rapeseed across China, the mapping task was divided into two subtasks: winter and spring rapeseed mapping (Fig. 2c). The planting boundaries for winter and spring rapeseed were determined by their growing seasons, with clearly different data collection times for the two planting areas.

### Landsat data

The Landsat program has provided over 50 years of moderate-resolution Earth observation images suitable for large-scale and long-term crop types mapping^42^. The GEE platform was used to access and process the surface reflectance data from November 1999 to September 2022 from four Landsat satellite sensors, including Landsat-5 Thematic Mapper (TM), Landsat-7 Enhanced Thematic Mapper Plus (ETM+), Landsat-8 Operational Land Imager (OLI), and Landsat-9 Operational Land Imager 2 (OLI-2). Images for the winter rapeseed growing region were gathered between November 1 of the preceding year and June 1 of the current year, while images for the spring rapeseed growing region were gathered between April 1 and September 1 of every year. To increase the observation frequency within the time series, multiple Landsat sensors were combined within overlapping periods. An inter-sensor harmonization approach was applied to eliminate the spectral differences between TM/ETM+ and OLI sensors^43,44^.

Several vegetation indices (VIs) were then calculated for each Landsat image. The normalized difference vegetation index (NDVI)^45^ and NDYI^27^ are commonly used due to their ability to detect time-series flowering signals^31,32^. The CI effectively captures variations in spectral reflectance of rapeseed during flowering and is commonly used for automatic rapeseed mapping^26^. The Winter Rapeseed Index (WRI) enhances the separability of winter rapeseed from winter crops^24^. Therefore, time-series NDVI, NDYI, CI, and WRI were combined to describe the growth status of rapeseed (Eqs. (1–4)):

1 $$NDVI=\frac{\rho NIR-\rho Red}{\rho NIR+\rho Red},$$

2 $$NDYI=\frac{\rho Green-\rho Blue}{\rho Green+\rho Blue},$$

3 $$CI=\rho NIR\times (\rho Red+\rho Green),$$

4 $$WRI=\frac{\rho NIR-\rho Green}{\rho NIR+\rho Green}\times \frac{\rho Blue}{\rho Green+\rho Red},$$

where ρNIR, ρRed, ρGreen, and ρBlue denote the surface reflectance of the near-infrared, red, green, and blue bands in the Landsat images.

### GLDAS data

GLDAS data from 1999–2022 was used to extract environmental variables that can characterize the phenological changes in rapeseed^46^. We selected ten variables that were closely related to vegetation growth as the input of the regression model. These variables included plant canopy surface water, canopy water evaporation, potential evaporation rate, soil moisture at 0–10 cm and 10–40 cm depths, soil temperature at 0–10 cm and 10–40 cm depths, accumulated air temperature, downward shortwave radiation, and total precipitation rate. The 3-h GLDAS data was composited into daily time series. For the winter rapeseed growing region, the time series from November 1 of the preceding year to February 1 of the current year was used, while for the spring rapeseed growing region, the time series from April 1 to July 1 of each year was used. Additionally, a latitude, longitude, and elevation grid with 0.25° spatial resolution was constructed as the base geographic elements. These environmental variables have been applied in monitoring the flowering phenology of winter rapeseed in southern China^21^.

### Land cover data

Cropland layers were extracted from land cover data sources including GlobeLand30 for the years 2000, 2010, and 2020^47,48^, and WorldCover 10 m v100 for 2020^49^. To align the spatial resolution with that of GlobeLand30, the ESA WorldCover product was downsampled to 30 m using the nearest neighbor method. A unified cropland mask layer was subsequently created by merging the cropland coverage from both GlobeLand30 and WorldCover v100 products. This layer was employed to eliminate non-cropland pixels from the final rapeseed maps.

### Terrain data

The 30 m Digital Elevation Model (DEM) data were used to create a mask layer for rapeseed resultant maps^50^. We used the DEM to calculate the slope and removed pixels of rapeseed that had a slope > 25° as these lands are banned in China due to the risk of soil erosion, especially in southern mountains^51^.

### Collected rapeseed samples

The rapeseed point collection version 2 (RPC-2) dataset was compiled using field surveys and visual interpretation of satellite images (Table S2). This dataset builds upon our previous research on rapeseed mapping in southern China from 2017–2021^21^. Here we expanded the dataset by manually labeling over 900,000 samples using a hexagonal grid and stratified sampling strategy (Fig. 1c,d)^52^ to avoid spatial autocorrelation of the samples. The RPC-2 dataset was constructed by utilizing historical Google Earth very-high-resolution (GE-VHR) images from all of China to fill gaps in field survey data. Specifically, these images were divided into numbers of equal-area hexagon grids with an area of 10 km^2^. For hexagonal grids that did not contain rapeseed fields, five points were randomly selected as nonrapeseed samples, while five to ten points for rapeseed fields were further collected for grids that contain rapeseed fields. The RPC-2 dataset covers over one million km^2^ and spans the period of 2000–2022, being a useful resource for large-scale and long-term rapeseed mapping.

### Sentinel-1 data

Time-series Sentinel-1 C-band SAR data spanning from 2014 to 2022 were used to rectify the flowering time of rapeseed as recorded in the RPC-2 dataset. Given the Sentinel-1 satellite’s capability for continuous, all-weather imaging and its high temporal resolution, we adopted the method proposed by d’Andrimont^6^ and Liu^21^ to identify peak rapeseed signals during the flowering period using the VV polarization of the Sentinel-1 data. The value of the VV polarization band reaches the peak with the peak-flowering period of rapeseed. We removed rapeseed samples with a time difference greater than five days by comparing the peak-flowering date of rapeseed obtained from the SAR data with the flowering date recorded in the RPC-2 dataset.

### Agricultural statistical data

We obtained information on the annual planted area of rapeseed from agricultural statistics yearbooks at the provincial and municipal levels in China. In cases where municipal-level data was not accessible, equivalent provincial data was used. Specifically, we collected data on the planted area of rapeseed for approximately 220 administrative regions from 2000 to 2021 and compared the results of satellite mapping with statistical data for consistency.

### Existing rapeseed products

To evaluate the accuracy and consistency of the CARM30 dataset, we compared it with two publicly available Chinese rapeseed products: (1) Liu’s REM product at 10 m resolution, which covered the Yangtze River Economic Belt from 2017 to 2021^21,53^; and (2) Zang’s rapeseed maps at 20 m resolution, which spanned the whole China from 2017 to 2021^22,54^.

### Monitoring rapeseed peak flowering phenology

**Fig. 3:**
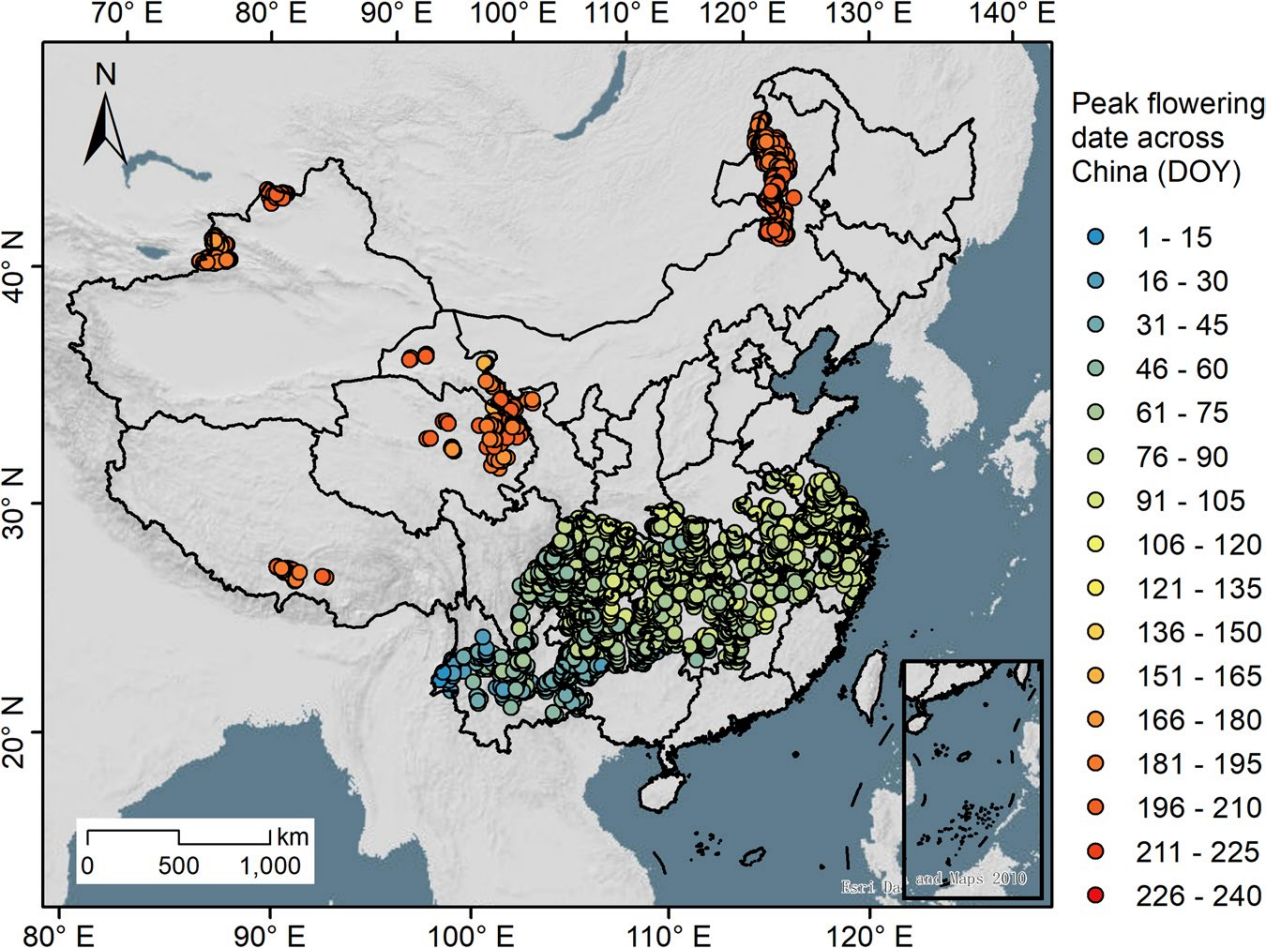
Distribution of training samples for the RFR model with peak flowering dates of rapeseed recorded (Julian day). DOY: day of year.

We used the RPC-2 dataset and the GLDAS environmental variables to study the flowering phenophase of rapeseed in China. A method proposed by Liu and Zhang^21^ for estimating rapeseed flowering phenology using the random forest regression (RFR) model and environmental variables was applied to map peak flowering dates. The RPC-2 dataset provided the training data, which were calibrated using time-series Sentinel-1 data and Whitaker smoothers^55,56^. Rapeseed samples with a time difference of five days were retained by comparing the flowering dates from both Sentinel-1 data and the RPC-2 dataset. To avoid model local overfitting, the calibrated training data were aggregated to a 0.25° spatial resolution and matched with GLDAS data, whose distribution is depicted in Fig. 3. The training data were predominantly located in the Yangtze River Basin, Xinjiang, Nei Mongol, and the Tibetan Plateau. Subsequently, we developed an RFR model to estimate the peak flowering date of rapeseed in China from 2000 to 2022. The model incorporated 13 time-series environmental variables, including 10 from GLDAS and factors such as latitude, longitude, and elevation. The *mtry* and *ntree* parameters of the model were set to the square root of the total number of input variables and 100. We used 3,687 training samples for the RFR model and an additional 1,580 independent samples to validate the performance of the RFR model. The R^2^ of the estimated peak flowering dates for winter rapeseed and spring rapeseed were 0.88 and 0.83, indicating the high accuracy and reliability of the model. (Fig. S1).

### Generating and optimizing training samples

**Fig. 4:**
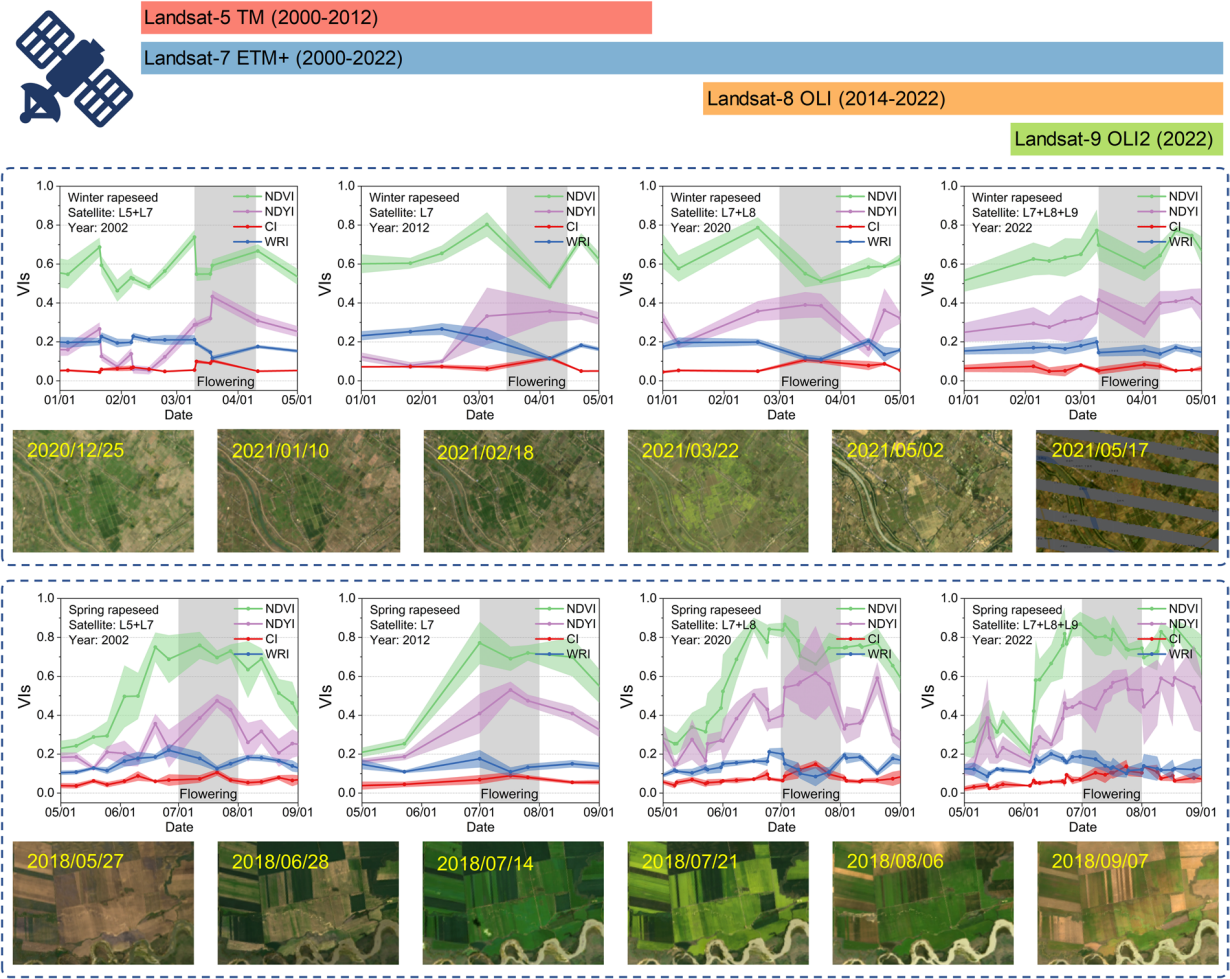
Temporal profiles of NDVI, NDYI, CI, and WRI of winter and spring rapeseed in China at different Landsat sensors and in different years. Subplots of each time-series profile were calculated from about 200 samples, collected from Jingzhou, Hubei (around 113.06943°E, 30.17444°N) and Yili, Xinjiang (around 80.7935°E, 42.9445°N), respectively. The mean values are depicted by lines, while the standard deviations are indicated as bands. The shaded areas indicate the flowering period of rapeseed. RGB composite maps were from Landsat images.

We automatically generated training samples for a machine learning classifier using a phenology-based rapeseed mapping approach to address the spatial imbalance of the manually collected samples. Four VIs were used to analyze the spectral and phenological patterns of rapeseed (Fig. 4). Due to the varying orbiting times of Landsat satellites, there were four satellite combination cases: L5+L7 (2000–2012), L7 (2013), L7+L8 (2014–2022), and L7+L8+L9 (2022). The flowering phenology of rapeseed was determined from the previous subsection and the estimated peak flowering dates of rapeseed are shown in Fig. S2. Winter rapeseed typically blooms in March, while spring rapeseed blooms in July. These VIs amplified the signal and enhanced the separability of rapeseed during flowering and can adaptively generate training samples for supervised classifiers.

Table 1 shows a statistical analysis evaluating the ability of four VIs to enhance the separability of rapeseed from other land covers under different Landsat combination scenarios. The t-value is the statistic which is calculated for comparing two phenophase pairs, while the p-value is the significance level. Higher values of |t| represent significant fluctuations of index values. For winter rapeseed, both CI and WRI performed well (p <0.01), indicating their ability to highlight rapeseed from pre-flowering to flowering stages. However, NDVI and NDYI had p > 0.1 in some scenarios, indicating that these two indices were less robust. WRI was less effective for spring rapeseed as it was designed for winter rapeseed. Overall, CI performed well in almost all Landsat combination scenarios, achieving the highest or second-highest t-values. More importantly, CI can identify rapeseed using only images during flowering, reducing its dependence on multi-temporal. Therefore, the CI-based identification method was adopted to generate initial machine-learning training samples.

**Table 1:** Results of statistical analysis for winter and spring rapeseed under different Landsat combination scenarios.

Pairs of VIs in various phenophase		L5+L7		L7		L7+L8		L7+L8+L9	
		|t|	p	|t|	p	|t|	p	|t|	p
Winter rapeseed	NDVI, pre-flowering (Jan and Feb) vs. flowering (Mar)	1.086	0.286	1.292	0.207	6.094	0	5.962	0
	NDYI, pre-flowering (Jan and Feb) vs. flowering (Mar)	24.727	0	**13.586**	0	5.049	0	0.735	0.467
	CI, pre-flowering (Jan and Feb) vs. flowering (Mar)	**34.342**	0	9.569	0	**16.526**	0	6.078	0
	WRI, pre-flowering (Jan and Feb) vs. flowering (Mar)	13.721	0	8.649	0	14.391	0	**7.107**	0
Spring rapeseed	NDVI, pre-flowering (May and Jun) vs. flowering (Jul)	31.791	0	9.977	0	9.854	0	5.688	0
	NDYI, pre-flowering (May and Jun) vs. flowering (Jul)	**35.429**	0	**21.989**	0	16.955	0	**16.781**	0
	CI, pre-flowering (May and Jun) vs. flowering (Jul)	7.762	0	16.993	0	**17.689**	0	13.773	0
	WRI, pre-flowering (May and Jun) vs. flowering (Jul)	4.829	0	1.184	0	0.728	0.352	3.796	0

Bolded numbers: highest performance. Underlined numbers: second-highest performance.

We calculated CI bands for each Landsat surface reflectance image collected during the flowering period according to the peak flowering time of rapeseed determined in the preceding section. These CI bands were classified into rapeseed and non-rapeseed categories by threshold segmentation (the segmentation threshold of the CI was set to 0.07 as suggested by Ashourloo, *et al*.^26^). Finally, we generated 2000 rapeseed and non-rapeseed initial samples respectively for each classification block with a stratified random sampling method.

To increase the purity of the training sample pool, the spectral angle mapper (SAM)^57–60^ was used to eliminate noisy samples. The reflectance spectrum of noisy samples generated by CI differs from that of the reference rapeseed endmember, making them distinguishable using spectral similarity measures. The spectral angle *α* for a pixel *i* can be calculated using the following arc cosine Eq. (5).

5 $$\alpha {=\cos }^{-1}\left(\frac{\mathop{\sum }\limits_{i=1}^{nb}{u}_{i}{r}_{i}}{\sqrt{\mathop{\sum }\limits_{i=1}^{nb}{u}_{i}}\sqrt{\mathop{\sum }\limits_{i=1}^{nb}{r}_{i}}}\right),$$

where *u* is the spectrum of unknown pseudo pixels, *r* is the spectrum of reference rapeseed, *nb* is the number of spectral channels, and the spectral angle *α* is the error metric of the SAM. An unknown pixel is classified as a noisy pixel if its spectral angle *α* with the reference rapeseed spectrum is higher than a determined threshold. In this study, we determined the threshold of the spectral angle as the standard deviation of the reference rapeseed spectrum in each subregion.

### Mapping rapeseed on GEE platform

The SARM method that combines the CI-based approach and multiple RF classifiers^61,62^ was implemented on GEE to map rapeseed across China^24^. This method assumes that for a pixel *i*, there are at least *m* cloud-free images available in a time series of *n* Landsat images with cloud contamination to support supervised classification. The results of the classification on cloud-free images are used to supplement the results of the cloud-contaminated images. The final classification of rapeseed is a combination of *m* probability maps of rapeseed, represented by Eq. (6).

6 $${P}_{i}=\frac{\mathop{\sum }\limits_{j=1}^{m}\,{p}_{i,j}}{m},$$

where *p*_*i,j*_ is the classification probability of pixel *i* in *j* temporal image, *m* is the number of cloud-free images, 1 ≤ *m* ≤ *n*. *P*_*i*_ is the probability that pixel *i* is finally classified into rapeseed. In the binary classification task of rapeseed and non-rapeseed, pixels with *P*_*i*_ > 50% are classified as rapeseed category.

Considering the variations in rapeseed cultivation patterns across China, we divided each mapping region into several 2° × 2° grids and trained local RF classifiers to minimize spatial heterogeneity. The *ntree* for each RF classifier was set to 100 and the *mtry* was set to the square root of the total number of input features. The input variables included Landsat monthly median composite images and derived VIs. Training samples for each grid were taken from adjacent 3 × 3 grids following a tile-based sampling strategy^52^. Additionally, 10-fold cross-validation was employed for RF classifiers to minimize uncertainty from potentially biased samples.

The classified rapeseed maps were optimized by removing non-cropland pixels and morphological post-processing to generate the CARM30 product. First, non-cropland pixels erroneously classified as rapeseed were eliminated using a unified cropland mask derived from GlobeLand30 and WorldCover v100, as shown in Table S3. These pixels represented 0.035% and 1.567% of the cropland and rapeseed pixels, respectively, potentially leading to an average annual mapping error of 111.022 k ha. Second, the bwareaopen function in MATLAB R2022a was used to fill holes in closed objects, preserving the integrity of the rapeseed fields. In northern China, rapeseed fields are large and regular while rapeseed fields are small and scattered in southern China. To account for these differences, the hole thresholds were set at 1 ha and 5 ha for winter rapeseed and spring rapeseed respectively.

## Data Records

The produced CARM30 dataset from 2000 to 2022 are available at Mendeley Data (10.17632/hxhkphgmtt.1)^63^. The format of these datasets is GeoTiff and the coordinate system is set to WGS 1984 UTM Zone 48 N. The values 0 and 1 of CARM30 represent non-rapeseed and rapeseed, respectively.

## Technical Validation

### Validation metrics

Four metrics were used for accuracy assessment, including user’s accuracy (UA), producer’s accuracy (PA), overall accuracy (OA), and F1 score. These metrics were derived from the confusion matrix, which can be measured by Eqs. (7–10):

7 $$UA=\frac{TP+TN}{N},$$

8 $$PA=\frac{TP}{TP+FP},$$

9 $$OA=\frac{TP+TN}{N},$$

10 $$F1=\frac{2\times UA\times PA}{UA+PA},$$

where *N* is the total number of validation samples, TP is the number of pixels correctly classified as rapeseed, TN is the number of pixels correctly as non-rapeseed, FP and FN refer to the number of pixels that are incorrectly classified as rapeseed and non-rapeseed, respectively.

Second, the CARM30 product was compared to two existing available rapeseed products. Third, the spatial consistency of the mapped rapeseed areas with agricultural statistics was measured. The evaluation metrics included coefficient of determination (R^2^), root mean square error (RMSE), and mean absolute error (MAE) (Eqs. (11–13)):

11 $${R}^{2}=\frac{{\left(\mathop{\sum }\limits_{i=1}^{n}({x}_{i}-\overline{{x}_{i}})\times ({y}_{i}-\overline{{y}_{i}})\right)}^{2}}{\mathop{\sum }\limits_{i=1}^{n}{({x}_{i}-\overline{{x}_{i}})}^{2}\times \mathop{\sum }\limits_{i=1}^{n}{({y}_{i}-\overline{{y}_{i}})}^{2}},$$

12 $$RMSE=\sqrt{\mathop{\sum }\limits_{i=1}^{n}\frac{{({x}_{i}-{y}_{i})}^{2}}{n}},$$

13 $$MAE=\frac{\mathop{\sum }\limits_{i=1}^{n}\left|{x}_{i}-{y}_{i}\right|}{n},$$

where *n* is the number of cities collected, *x*_*i*_ is the rapeseed mapped area for city *i* from satellite imagery, *y*_*i*_ is the statistical rapeseed area for city *i*, $\overline{{x}_{i}}$ and $\overline{{y}_{i}}$ are the corresponding mean values, respectively.

### Comparison with existing rapeseed maps

**Fig. 5:**
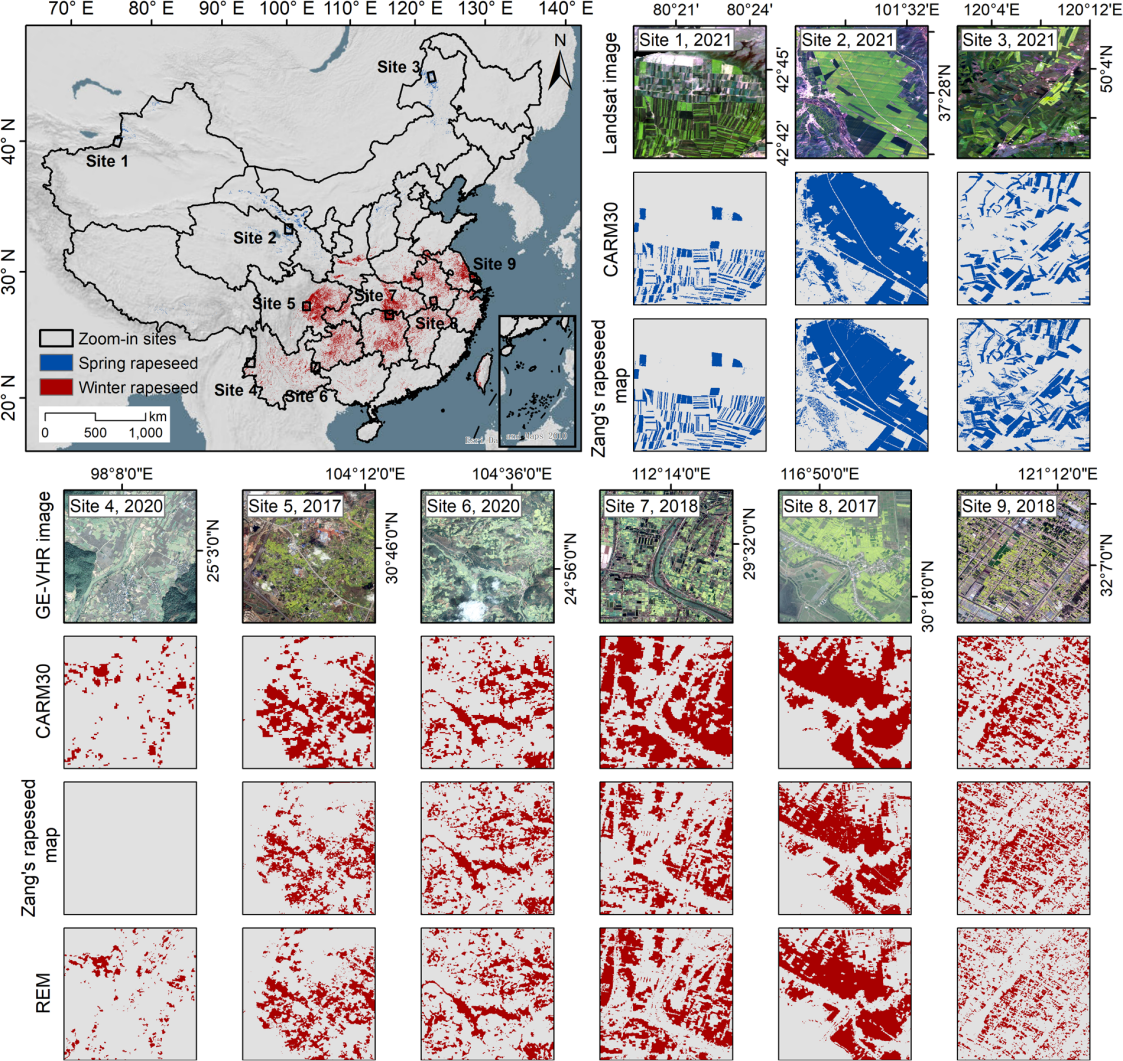
Inter-comparison of CARM30, Zang’s rapeseed map, and REM from 2017 to 2021. The benchmark year is 2021. The base satellite images are from Landsat (sites 1–3) and GE-VHR images (sites 4–9).

A qualitative assessment was conducted to compare CARM30 to 10 m REM and Zang’s 20 m rapeseed maps from 2017–2021. As depicted in Fig. 5, winter and spring rapeseed are represented by red and blue pixels. Six and three zoom-in views were selected for winter and spring rapeseed to compare the mapping details using satellite images. CARM and REM were only compared in southern China due to the absence of spring rapeseed in the REM dataset. Overall, CARM30 demonstrated high agreement with existing maps and accurately depicted the spatial distribution of rapeseed fields in both well-regularized northern China and scattered southern China. However, some discrepancies were observed (i.e., sites 7 and 8). Rapeseed fields described in CARM30 appeared more misclassified due to the coarser spatial resolution of Landsat imagery. This difference in satellite sensors has impacted the accuracy of the proposed method in southern China (as illustrated in Table 2). Nevertheless, CARM30 captured some omissions present in Zang’s map (as shown in site 4). In conclusion, our CARM30 product depicted the detailed distribution of rapeseed across China and maintained high consistency with existing rapeseed maps obtained from Sentinel data.

**Table 2:** Accuracy of CARM30 for winter and spring rapeseed from 2000 to 2022.

Year	Winter rapeseed				Spring rapeseed			
	UA	PA	OA	F1 score	UA	PA	OA	F1 score
2000	0.866	0.958	0.944	0.909	0.975	0.962	0.982	0.968
2001	0.863	0.539	0.857	0.663	0.980	0.958	0.982	0.969
2002	0.973	0.546	0.800	0.699	0.992	0.953	0.984	0.972
2003	0.981	0.851	0.943	0.912	0.997	0.930	0.979	0.962
2004	0.947	0.854	0.974	0.898	0.997	0.982	0.994	0.989
2005	0.995	0.887	0.937	0.938	0.985	0.982	0.991	0.983
2006	0.939	0.917	0.936	0.928	0.995	0.989	0.995	0.992
2007	0.935	0.858	0.969	0.895	0.993	0.992	0.996	0.992
2008	0.714	0.841	0.968	0.773	0.998	0.885	0.967	0.938
2009	0.962	0.885	0.959	0.922	0.989	0.989	0.993	0.989
2010	0.949	0.885	0.944	0.916	0.993	0.983	0.993	0.988
2011	0.934	0.886	0.943	0.910	0.993	0.979	0.992	0.986
2012	0.931	0.789	0.937	0.854	0.994	0.903	0.971	0.946
2013	0.940	0.657	0.909	0.773	0.994	0.956	0.986	0.975
2014	0.946	0.884	0.945	0.914	0.990	0.957	0.985	0.974
2015	0.940	0.926	0.961	0.933	0.972	0.976	0.985	0.974
2016	0.913	0.959	0.970	0.936	0.982	0.963	0.984	0.972
2017	0.912	0.950	0.967	0.930	0.931	0.969	0.971	0.950
2018	0.910	0.882	0.955	0.896	0.989	0.941	0.980	0.964
2019	0.846	0.796	0.930	0.820	0.984	0.963	0.985	0.973
2020	0.921	0.883	0.949	0.902	0.972	0.976	0.985	0.974
2021	0.754	0.975	0.934	0.850	0.988	0.936	0.978	0.961
2022	0.699	0.985	0.907	0.818	0.981	0.920	0.972	0.950
Average	0.903	0.852	0.936	0.869	0.985	0.958	0.984	0.971

### Pixel-wise validation using ground reference data

**Fig. 6:**
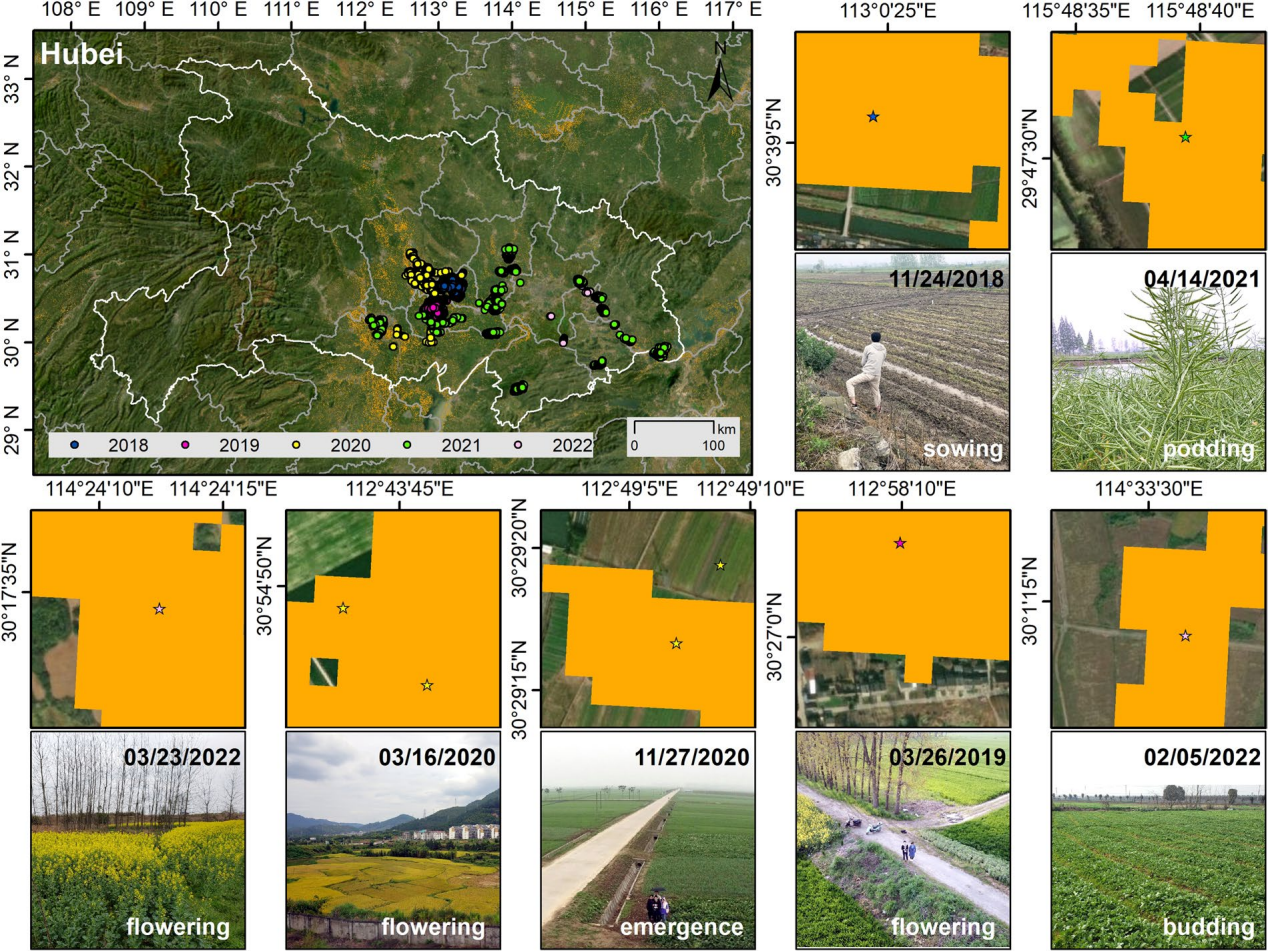
Fieldworks and corresponding field photos in Hubei Province, China. Rapeseed pixels are shown in orange. The base satellite imagery is from the ESRI © community.

The accuracy of the CARM30 product was qualitatively evaluated using ground reference data. This dataset comprises field survey data and sample points interpreted visually from GE-VHR imagery, with the field survey data and corresponding photos illustrated in Fig. 6. Fieldwork conducted in Hubei Province of China between 2018 and 2022 provided land cover sample data, including rapeseed. The sub-maps in Fig. 6 indicate a strong spatial correlation between CARM30 and the field survey data, accurately representing rapeseed field types. However, the delineation of rapeseed field boundaries remains imprecise due to the coarse spatial resolution of the Landsat image.

The accuracy of the CARM30 product from 2000 to 2022 was verified using RPC-2 ground samples, as presented in Table 2. Overall, CARM30 demonstrated high precision with F1 scores exceeding 0.8 for most years. The accuracy of winter rapeseed was significantly lower than that of spring rapeseed, with average F1 scores of 0.869 and 0.971. In 2001 and 2002, the precision of winter rapeseed was relatively poor with F1 scores below 0.7. In contrast, the accuracy of spring rapeseed remained consistently high throughout all years with F1 scores above 0.9.

We further evaluated the performance of Zang’s rapeseed maps using the same validation data, as shown in Table 3. In the winter rapeseed growing area, Zang’s product showed comparable performance to CARM30, with an average F1 score of 0.877 (CARM: 0.88). However, in the spring rapeseed growing area, Zang’s product underperformed compared to CARM30, with an average F1 score of 0.899 (CARM30: 0.964). Both CARM30 and Zang’s product exhibited lower validation accuracy for winter rapeseed compared to spring rapeseed.

**Table 3:** Accuracy of the Zang’s product for winter and spring rapeseed from 2017 to 2022.

Year	Winter rapeseed				Spring rapeseed			
	UA	PA	OA	F1 score	UA	PA	OA	F1 score
2017	0.956	0.827	0.952	0.887	0.953	0.882	0.954	0.916
2018	0.973	0.831	0.958	0.896	0.992	0.890	0.966	0.938
2019	0.975	0.648	0.926	0.779	0.988	0.923	0.975	0.955
2020	0.947	0.861	0.950	0.902	0.968	0.871	0.955	0.917
2021	0.904	0.937	0.969	0.920	0.972	0.636	0.891	0.768
Average	0.951	0.821	0.951	0.877	0.974	0.840	0.948	0.899

Several factors contribute to the relatively poor performance of winter rapeseed compared to spring rapeseed. First, the topographical complexity in southern China results in extreme fragmentation of cropland, with most fields measuring less than 0.04 ha^64,65^. This has led to confusion between rapeseed and other land cover types^66^. Second, smallholder agriculture in southern China exhibits more complex cultivation patterns than intensive farm operations in northern China^67^. The arbitrary intercropping patterns result in high spatial heterogeneity of cropland, affecting the accuracy of classification algorithms. Lastly, the unavailability of high-quality Landsat images introduces uncertainty to the produced rapeseed maps. Cloudy and rainy conditions in southern China result in fewer high-quality satellite images being available than in northern China^68^, posing challenges for rapeseed mapping.

### Comparison with agricultural statistics

**Fig. 7:**
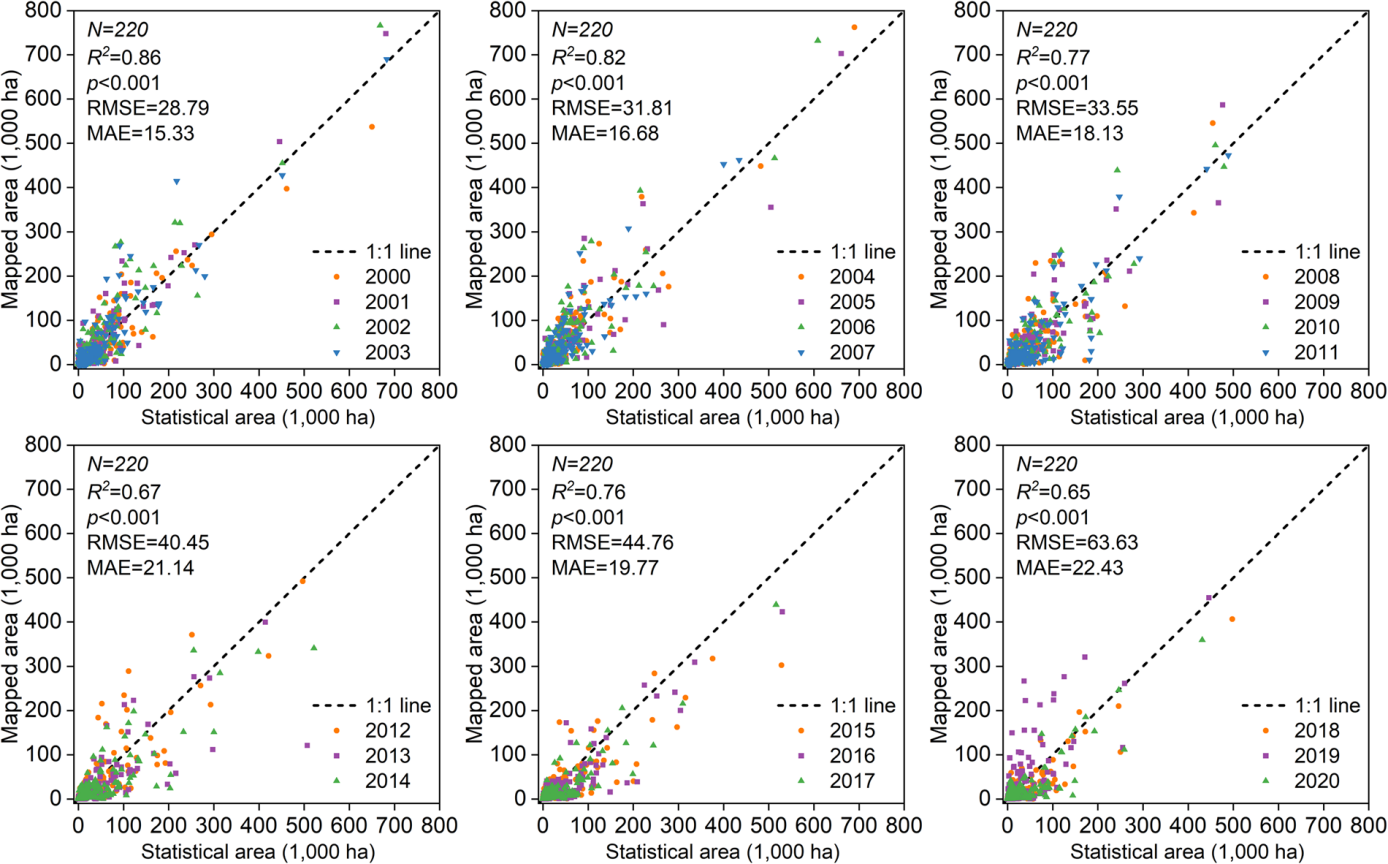
Linear regression of CARM30-derived rapeseed planting areas with statistics at the municipal level.

The CARM30 product was compared with statistics for the years 2000 to 2020. As depicted in Fig. 7, the mapped rapeseed area from CARM30 showed a strong correlation with agricultural statistics with an R^2^ ranging from 0.65 to 0.86 (*p* <0.001). According to RMSE and MAE, the two data sources differed by 28.79 to 63.63 k ha and 15.33 to 22.43 k ha, respectively. In earlier years, CARM30 showed a high association with statistical data. The remotely sensed rapeseed planting area, however, has been smaller than the statistical area since 2012, resulting in a lower R^2^ and higher RMSE and MAE.

**Fig. 8:**
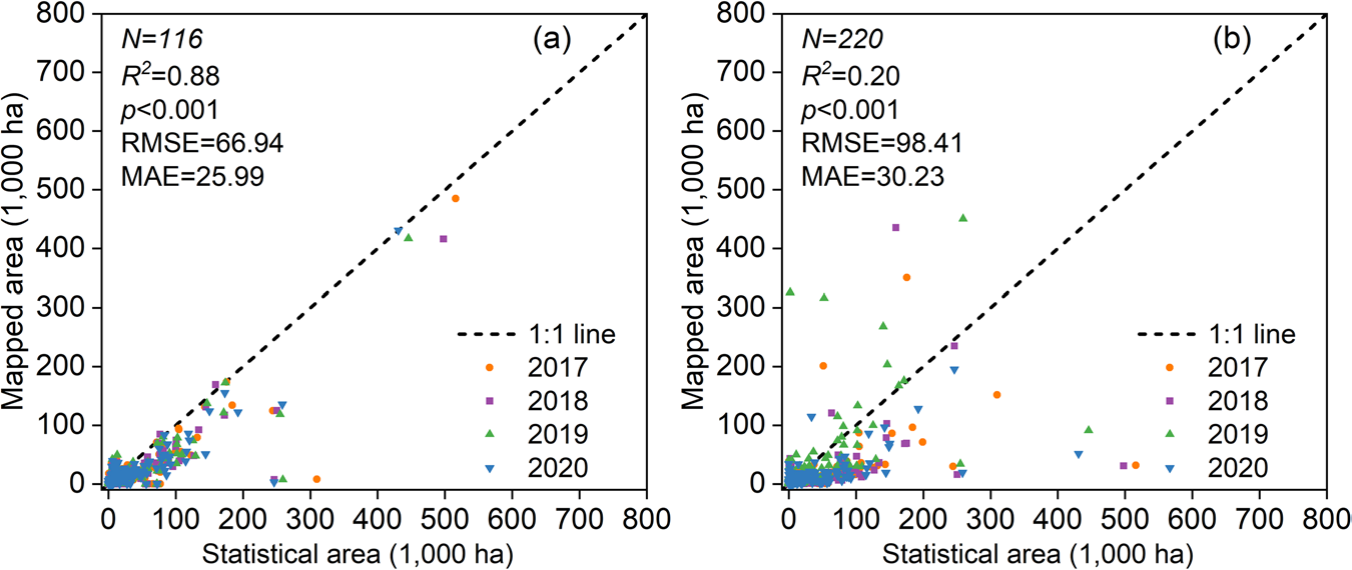
Linear regression of rapeseed areas calculated from two existing rapeseed products with statistics at the municipal level. (**a**) REMs, (**b**) Zang’s rapeseed maps.

The REM dataset and Zang’s rapeseed maps were further compared with agricultural statistics (Fig. 8). Even though REM had a high R^2^, it nevertheless underestimated the officially reported rapeseed area (Fig. 8a). RMSE and MAE for Zang’s rapeseed map were 98.41 k ha and 30.23 k ha, respectively, with an R^2^ of 0.2 (Fig. 8b). The substantial discrepancies between statistics and the remotely sensed rapeseed area in recent years may be due to changes in the market and in policy. Rapeseed is a labor-intensive crop with high costs and low returns^10^. Agricultural producers in China are choosing more cost-effective crops as a result of the country’s urbanization and industrialization, which have reduced the manpower resources available for agricultural cultivation^69,70^. Additionally, inaccurate figures may result from farmers overstating the rapeseed planted area due to economic factors like agricultural subsidies^71,72^.

### Spatial patterns of rapeseed cultivation in China

**Fig. 9:**
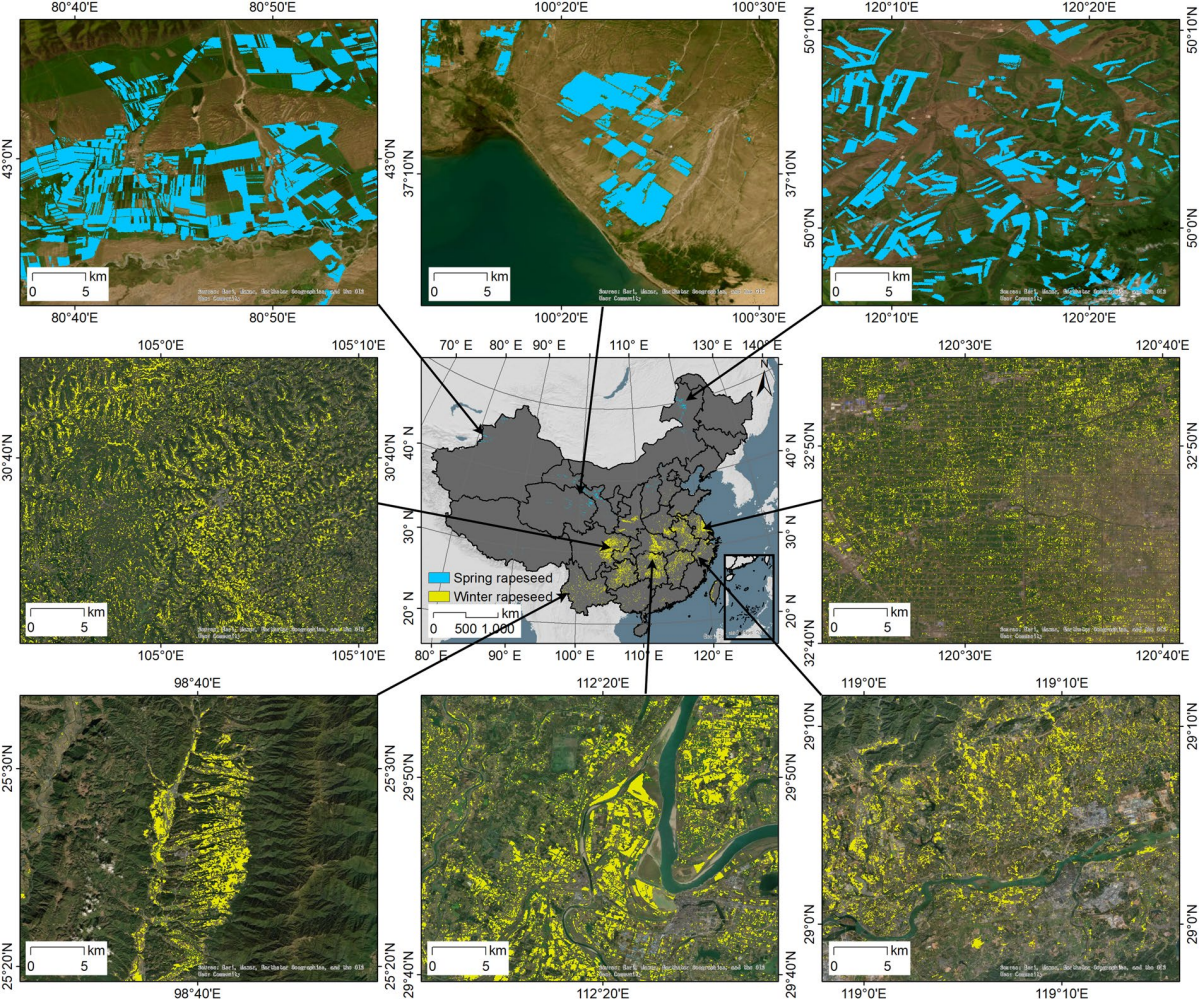
Spatial details of rapeseed fields across China. The benchmark year is 2021. Spring rapeseed and winter rapeseed are displayed in blue and yellow pixels. The base satellite imagery is from the ESRI © community.

Figure 9 depicts the spatial distribution and field patterns of 30 m rapeseed across various regions in China. In northern China, spring rapeseed fields are large and regularly shaped due to large-scale intensive management. In contrast, winter rapeseed fields are fragmented and irregular, primarily found in central China (i.e., Sichuan, Guizhou, Hubei, Hunan, and Jiangxi). Field patterns vary with the topography. In mountainous southwest China, rapeseed is concentrated in narrow valleys. While in the central region, rapeseed is fragmented by the river network. In Jiangsu and Anhui, small-scale household cultivation has resulted in a dense and spotty distribution of rapeseed fields.

**Fig. 10:**
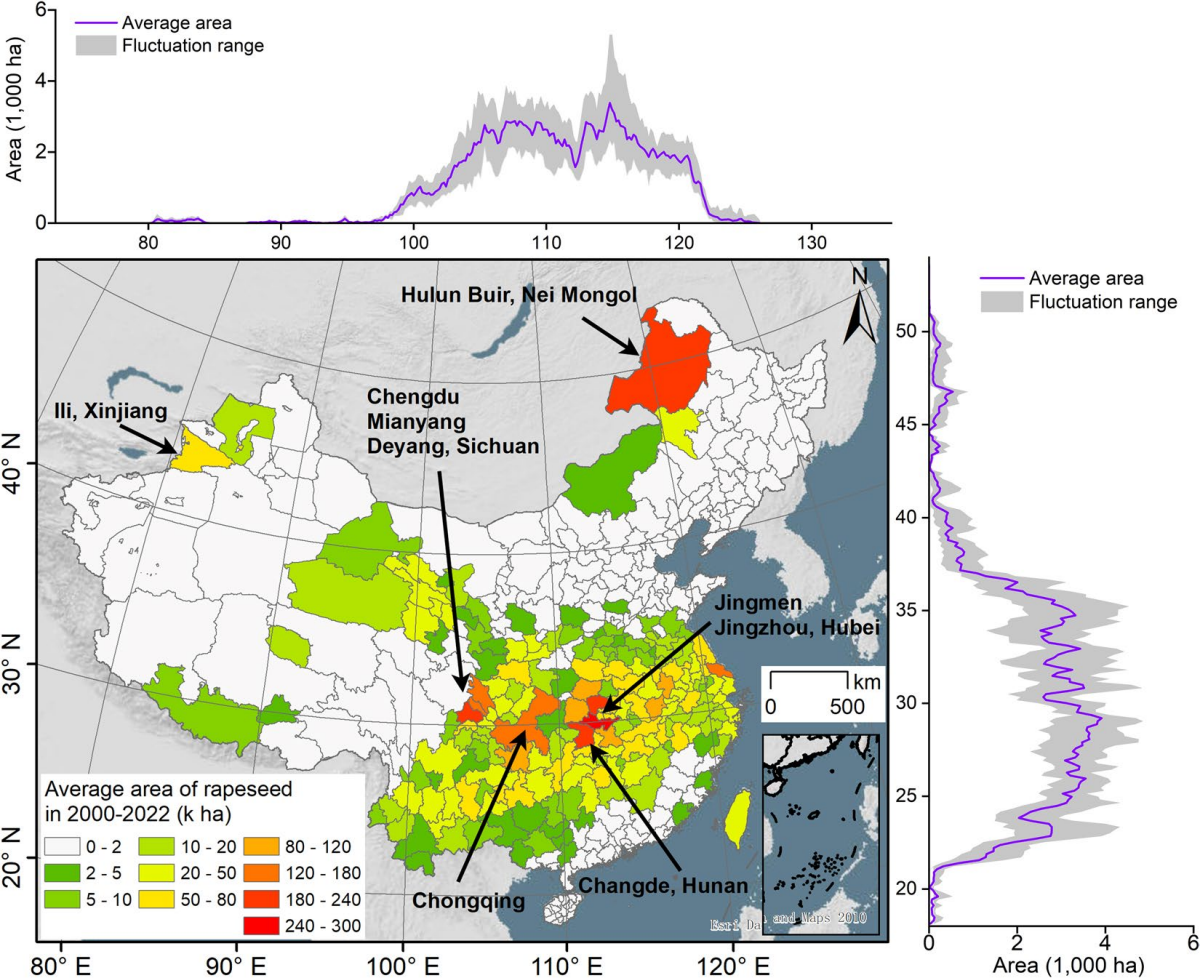
Spatial patterns of rapeseed across latitudinal, longitudinal, and municipal level. The purple line indicates the average acreage of rapeseed from 2000 to 2022, and the gray band represents the fluctuation range of rapeseed area over 23 study years.

The spatial distribution of rapeseed at different latitudes, longitudes, and city-level scales was further analyzed and visualized using the CARM30 product (Fig. 10). Rapeseed in China is primarily distributed between 100°E–122°E and 21°N–37°N. Spring rapeseed is mainly found in Yili and Hulunbeier, while winter rapeseed is primarily distributed in cities near 30°N (i.e., Chengdu, Chongqing, Jingmen, and Changde). The purple curve represents the average planting area of rapeseed from 2000 to 2022, while the gray strip indicates the fluctuation in the planting area of rapeseed. The significant range fluctuation suggests that the planting area of rapeseed has undergone major changes since the 21st century.

**Fig. 11:**
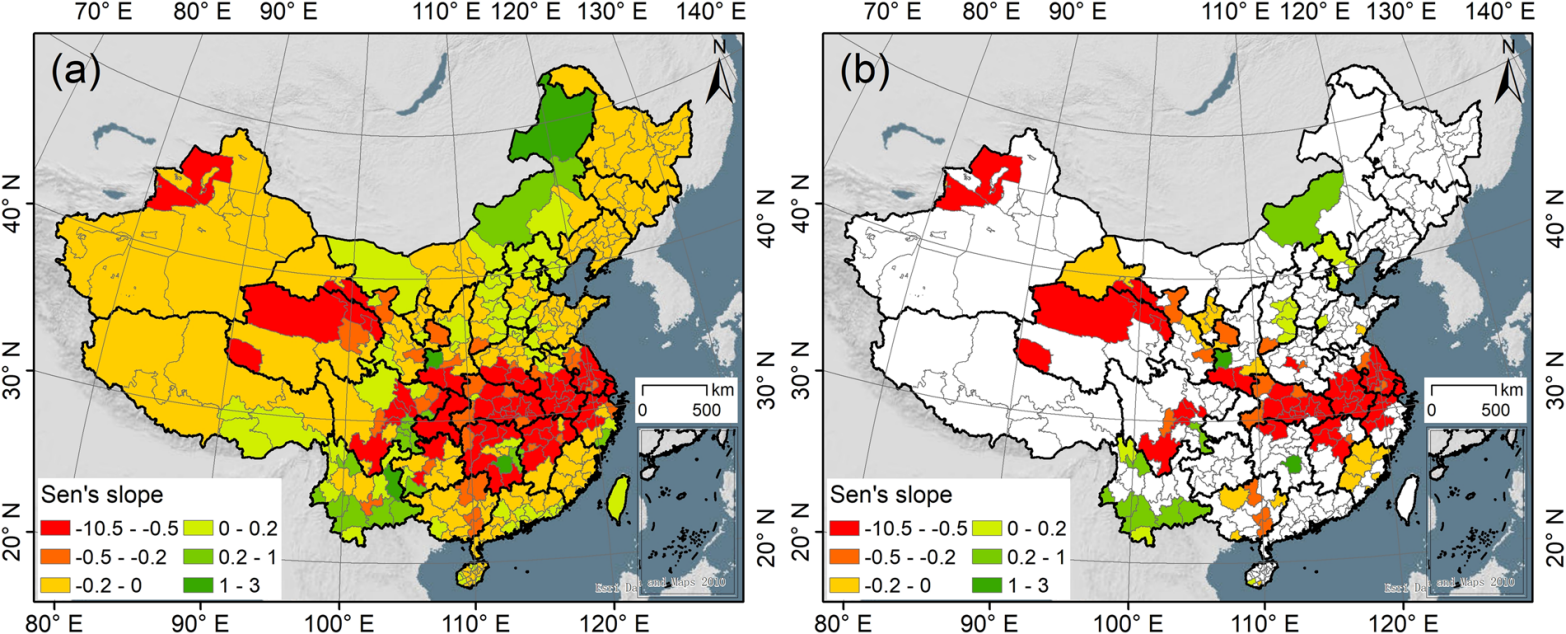
The dynamic trend of rapeseed planting area in China from 2000 to 2022. (**a**) Sen’s slope at the municipal level; (**b**) Sen’s slope at a 5% significance level.

The temporal trend of rapeseed cultivation from 2000 to 2022 was characterized using Sen’s slope analysis^73^, as depicted in Fig. 11. China’s rapeseed planting area has exhibited a clear decreasing trend since the 21st century (decrease: 199 cities; increase: 94 cities). The most decreased areas in rapeseed production were primarily Sichuan, Chongqing, Hubei, Hunan, Anhui, Jiangxi, and Jiangsu. In contrast, the areas with increased rapeseed production were mainly Nei Mongol and Yunnan. Overall, the number of municipalities that detected a decrease and an increase in rapeseed production was 95 and 23, respectively (at a significance level of 5%).

## Usage Notes

### Advantages of our rapeseed maps

This study represents the inaugural large-scale mapping of rapeseed across China from 2000 to 2022. An initial collection of approximately 910,000 samples, both rapeseed and non-rapeseed, was conducted at a national scale. These samples were utilized to estimate the peak flowering dates of rapeseed and to assess the accuracy of the obtained CARM30 product. A Landsat image-based strategy was subsequently implemented for sample generation and purification, thereby providing training samples for the SARM algorithm. The CI-based rapeseed mapping approach was employed to automatically generate training samples, while the SAM algorithm was utilized to filter out noisy samples. The procured data is publicly accessible, facilitating researchers in investigating the spatial-temporal dynamics and phenological shifts of rapeseed in China.

This study presents several notable advantages. First, the RPC-2 dataset and an RFR model were employed to estimate the flowering period of rapeseed in China, addressing the issue of phenological variability. This approach enabled the estimation of a long time series of flowering phenology information for rapeseed nationwide, compensating for the previously unknown phenology of spring rapeseed^21^. The resulting rapeseed flowering maps have potential applications in yield estimation studies. Second, the scarcity of training samples on a national scale was addressed by employing an unsupervised CI-based mapping method and SAM to automatically generate and purify training samples. This approach, previously applied to rapeseed mapping in southern China^21^, was adapted to accommodate the temporal and spatial resolution of Landsat images. Multiple spectral and phenological indices were analyzed and compared, leading to the selection of the CI-based method for sample generation. The SAM method was employed to purify the samples, mitigating the impact of noise. The proposed process offers valuable technical insights for other crop mapping tasks. To our knowledge, CARM30 is the longest-period rapeseed dataset currently accessible. Results from both qualitative and quantitative assessments demonstrated that CARM30 is accurate and competitive with other 10 m and 20 m resolution rapeseed maps.

Specifically, CARM30 provides a wider temporal window than both REM and Zang’s rapeseed maps, allowing the tracking of rapeseed planting trends in China since the 21st century. Furthermore, compared to Zang’s rapeseed maps, CARM30 displays a higher association with statistical data and provides a wider spatial coverage than the REM dataset.

### Uncertainties

Despite the advantages achieved, there are still some uncertainties in CARM30. The fragmentation of cultivated land affects the accuracy of rapeseed mapping. In southern China, smallholder-operated croplands are often less than 0.07 ha in size, smaller than the size of a single Landsat pixel, making it challenging to differentiate between rapeseed and other crops^67^. One potential solution to this issue is to utilize Sentinel-2 data to create 10 m Landsat image products^74,75^. The availability of Landsat observations can also have an impact on CARM30 accuracy in some regions with heavy cloud cover.

**Fig. 12:**
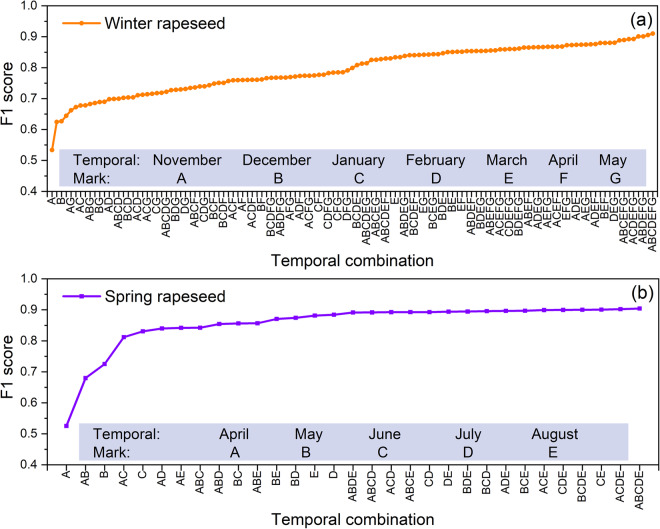
Variation in rapeseed mapping accuracy under different monthly image combinations (with the metric of F1 score).

To understand the uncertainty caused by the availability of Landsat images, we evaluated the variation in the accuracy of CARM30 with different monthly image combinations (Fig. 12). We utilized seven and five monthly compositions of images for the winter and spring rapeseed mapping tasks, resulting in 127 and 31 image combination scenarios, respectively. The lowest classification accuracy for rapeseed was obtained when only one monthly composited image from the sowing stage was used, while the highest accuracy was achieved when all monthly images were used. Compared to spring rapeseed, the classification accuracy of winter rapeseed improved gradually with an increasing number of images, indicating the complexity of the winter rapeseed mapping task.

**Fig. 13:**
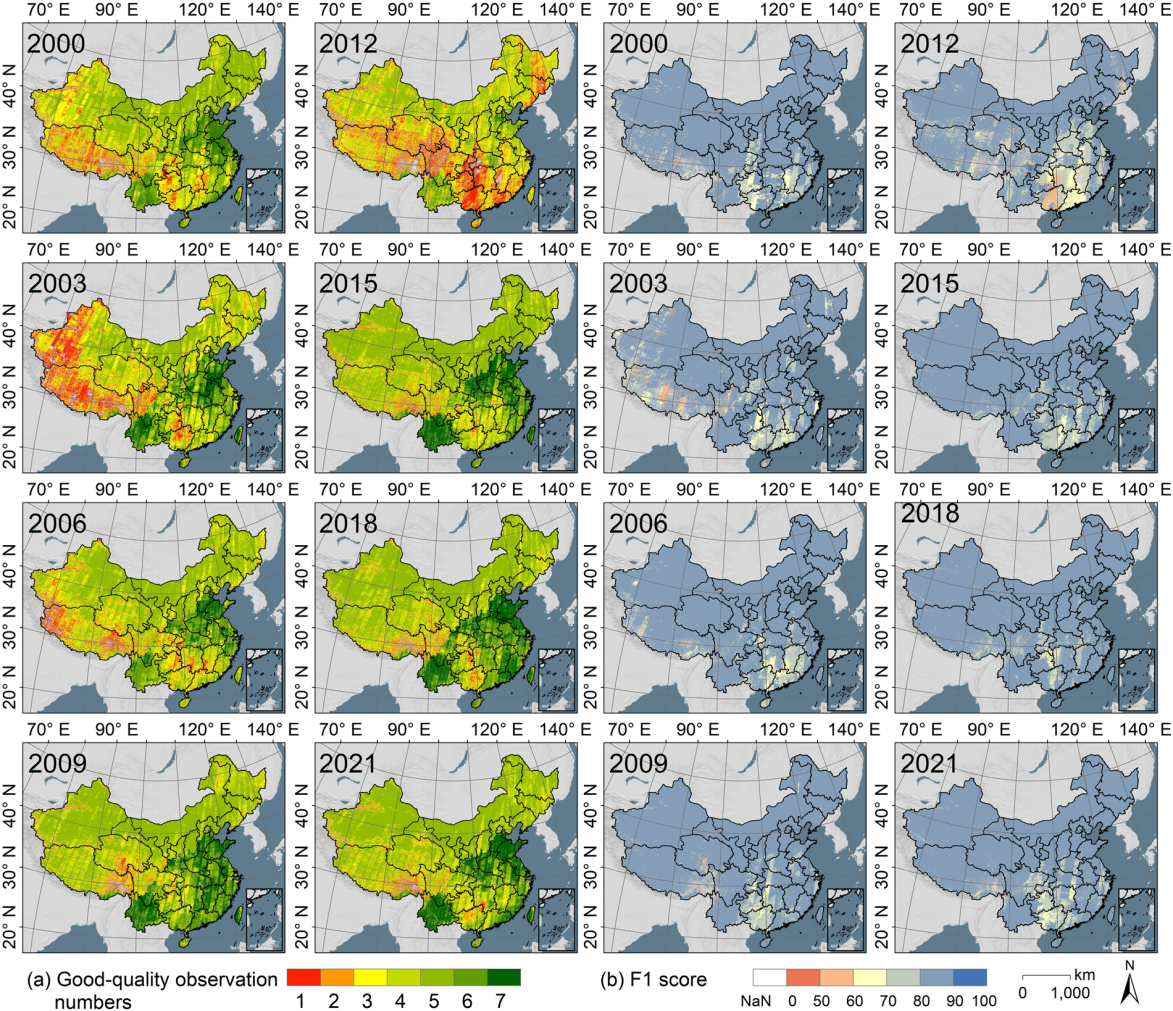
Distribution of the number of high-quality Landsat monthly composited images and the F1 scores for CARM30 across China during 2000 to 2022.

We visualized the impact of different image combinations on rapeseed mapping in China. Figure 13 illustrates the number of cloud-free Landsat monthly composited images (Fig. 13a) and the corresponding F1 scores for CARM30 during the rapeseed growing season (Fig. 13b). Spatially, the highest errors in CARM30 were found in southern and southwestern China (i.e., Guangxi, Guangdong, Hunan, Jiangxi, and Tibet). These regions often experience cloudy weather, leading to a lack of high-quality Landsat images and introducing uncertainty to CARM30. Temporarily, CARM30’s potential uncertainty peaked in 2012, the year that only Landsat-7 images were employed. However, the uncertainty in CARM30 due to data availability has decreased in recent years as a result of the expansion of Landsat satellites.

**Fig. 14:**
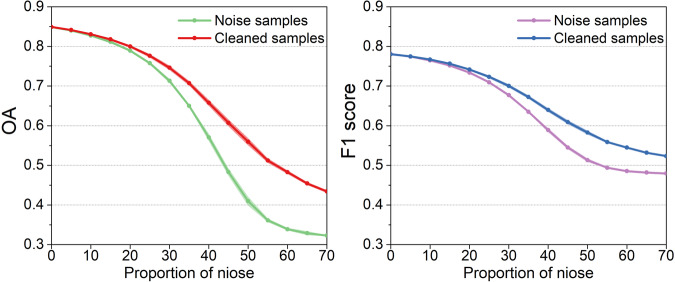
Accuracy changes of we proposed method under different proportion of noise in the CI-derived training samples. The mean values are depicted by lines, while the standard deviations are indicated as bands.

The uncertainty regarding the quality of training samples in the CARM30 dataset was further examined through a simulation experiment, with the test region depicted in Fig. S3. Figure 14 illustrates the contribution of the training sample cleaning strategy in enhancing accuracy under varying noise intensities. Observations indicate that the classification accuracy of the proposed method inversely correlates with the noise level in the CI-derived training samples. It suggests that using uncleaned training samples may result in high classification errors. The SAM method improves the purity of the initially generated samples and yields a higher classification accuracy, thereby demonstrating the effectiveness of our mapping strategy.

### Supplementary information

SUPPLEMENTARY INFORMATION

## Data Availability

The core codes and associated files we used for mapping rapeseed flowering phenology and spatial distribution are available at https://github.com/liuwenbinwhu/China-annual-rapeseed-maps30. Moreover, MATLAB R2022a, ArcGIS Pro, and Origin 2017 were used for data pre-processing, spatial analysis, and figure production.
