# Supplementary material for: CARM30: China annual rapeseed maps at 30 m spatial resolution from 2000 to 2022 using multi-source data
Source: Sci Data. 2024 Apr 8;11:356. doi: 10.1038/s41597-024-03188-1 (PMC11001952; doi:10.1038/s41597-024-03188-1)
Supplement: Supplementary file 1 — SUPPLEMENTARY INFORMATION [file 41597_2024_3188_MOESM1_ESM.pdf]

Contents

Table S1. 2
Table S2. 3
Table S3. 4
Fig.S1 5
Fig.S2 6
Fig.S3 7

Table S1. The multi-source dataset used in this study.

Data	Name	Time	Spatial and temporal resolution	Usage	Sources
Optical imagery	Landsat TM	1999–2012	30 m, 16 d	Analyzing the spectral characteristics and mapping spatial distribution of rapeseed	https://developers.google.com/earth-engine/datasets/catalog/landsat
	Landsat ETM+	1999–2022			
	Landsat OLI	2013–2022			
	Landsat OLI2	2021–2022			
RPC-2	RPC-1 dataset	2017–2021	0.6–30 m, yearly	Training model and validating accuracy	Collected dataset
	Manually labeled samples	2000–2022			
SAR imagery	Sentinel-1	2014–2022	10 m, 6 d	Calibrating the flowering time of rapeseed	https://developers.google.com/earth-engine/datasets/catalog/COPERNICUS_S1_GRD
GLDAS data	GLDAS-2.0	1999–2000	0.25 °, 3 h	Estimating the peak flowering phenology of rapeseed	https://developers.google.com/earth-engine/datasets/catalog/NASA_GLDAS_V20_NOAH_G025_T3H
	GLDAS-2.1	2000–2022			https://developers.google.com/earth-engine/datasets/catalog/NASA_GLDAS_V021_NOAH_G025_T3H
Land cover maps	GlobeLand30	2000, 2010, 2020	30 m, yearly	Masking non-cultivated land	http://www.globallandcover.com/home_en.html
	WorldCover	2020	10 m, yearly		https://developers.google.com/earth-engine/datasets/catalog/ESA_WorldCover_v100
Terrain data	SRTM DEM	2000	30 m	Removing pixels with a slope > 25°	https://developers.google.com/earth-engine/datasets/catalog/USGS_SRTMGL1_003
Statistical data	Agricultural Statistical Yearbook of each province and city	2000–2020	Provincial or municipal level, yearly	Comparing satellite-derived rapeseed maps	Statistical bureaus of provinces and cities in China
Existing rapeseed maps	REM dataset	2017–2021	10 m, yearly	Comparing spatial consistency among rapeseed maps	https://data.mendeley.com/datasets/6p6b86bwv5/2
	Zang's rapeseed maps	2017–2021	20 m, yearly		https://doi.org/10.5281/zenodo.7047270

Table S2. Detailed information about the RPC-2 dataset.

Year	Sampled area (km²)	Winter rapeseed growing region		Spring rapeseed growing region		Total
		Rapeseed	Non-rapeseed	Rapeseed	Non-rapeseed	
2000	6,026	1,578	3,763	4,000	10,000	19,341
2001	5,476	501	1,418	4,000	10,000	15,919
2002	8,572	1,012	1,326	4,000	10,000	16,338
2003	7,019	1,204	2,263	4,000	10,000	17,467
2004	6,491	294	1,900	4,000	10,000	16,194
2005	5,622	1,633	1,403	4,000	10,000	17,036
2006	5,857	1,274	1,590	4,000	10,000	16,864
2007	5,697	267	1,452	4,000	10,000	15,719
2008	5,807	107	1,542	4,000	10,000	15,649
2009	26,723	4,805	12,596	4,000	10,000	31,401
2010	26,295	6,192	11,850	4,000	10,000	32,042
2011	13,423	2,539	5,386	4,000	10,000	21,925
2012	45,471	6,819	22,421	4,000	10,000	43,240
2013	53,067	8,416	27,181	4,000	10,000	49,597
2014	95,226	18,923	38,214	4,000	10,000	71,137
2015	68,460	13,997	33,239	4,000	10,000	61,236
2016	57,879	8,595	29,715	4,000	10,000	52,310
2017	89,568	13,313	44,826	4,000	10,000	72,139
2018	140,710	20,568	74,166	4,000	10,000	108,734
2019	107,276	13,142	54,693	4,000	10,000	81,835
2020	67,744	12,353	34,057	4,000	10,000	60,410
2021	76,177	5,863	24,548	4,000	10,000	44,411
2022	90,473	4,216	15,654	4,000	10,000	33,870
Total	1,015,059	147,611	445,203	92,000	230,000	914,814

Table S3. Proportion and area of non-cropland pixels removed by the cropland mask.

Year	Proportion of non-cropland pixels to cropland pixels (%)	Proportion of non-cropland pixels to rapeseed pixels (%)	Area of non-cropland pixels (k ha)
2000	0.046	1.711	147.103
2001	0.024	0.899	78.677
2002	0.073	2.495	235.479
2003	0.035	1.278	111.657
2004	0.044	1.520	140.378
2005	0.034	1.303	107.877
2006	0.044	1.525	141.029
2007	0.027	1.173	87.165
2008	0.037	1.658	117.717
2009	0.018	0.731	57.823
2010	0.017	0.693	55.844
2011	0.050	2.056	160.544
2012	0.064	2.580	205.830
2013	0.039	2.037	123.771
2014	0.032	1.635	102.700
2015	0.019	0.989	60.344
2016	0.033	1.816	105.240
2017	0.036	2.376	116.686
2018	0.036	2.315	115.535
2019	0.031	1.117	100.847
2020	0.009	0.597	27.505
2021	0.014	0.964	44.630
2022	0.034	2.585	109.115
Average	0.035	1.567	111.022

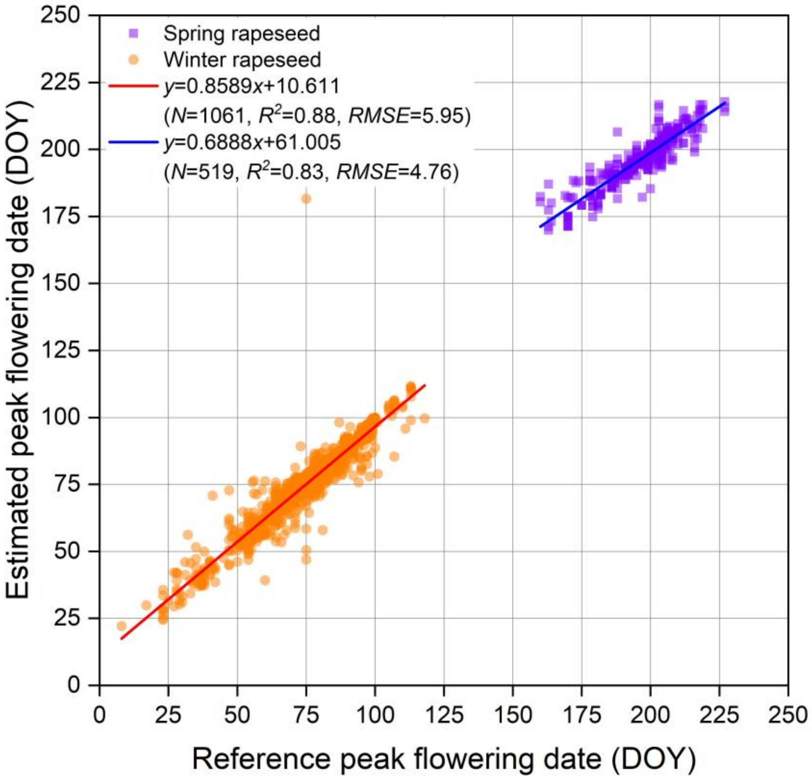

Fig.S1 Linear relationship between reference peak flowering date and estimated peak flowering date from the RFR model. DOY: day of year.

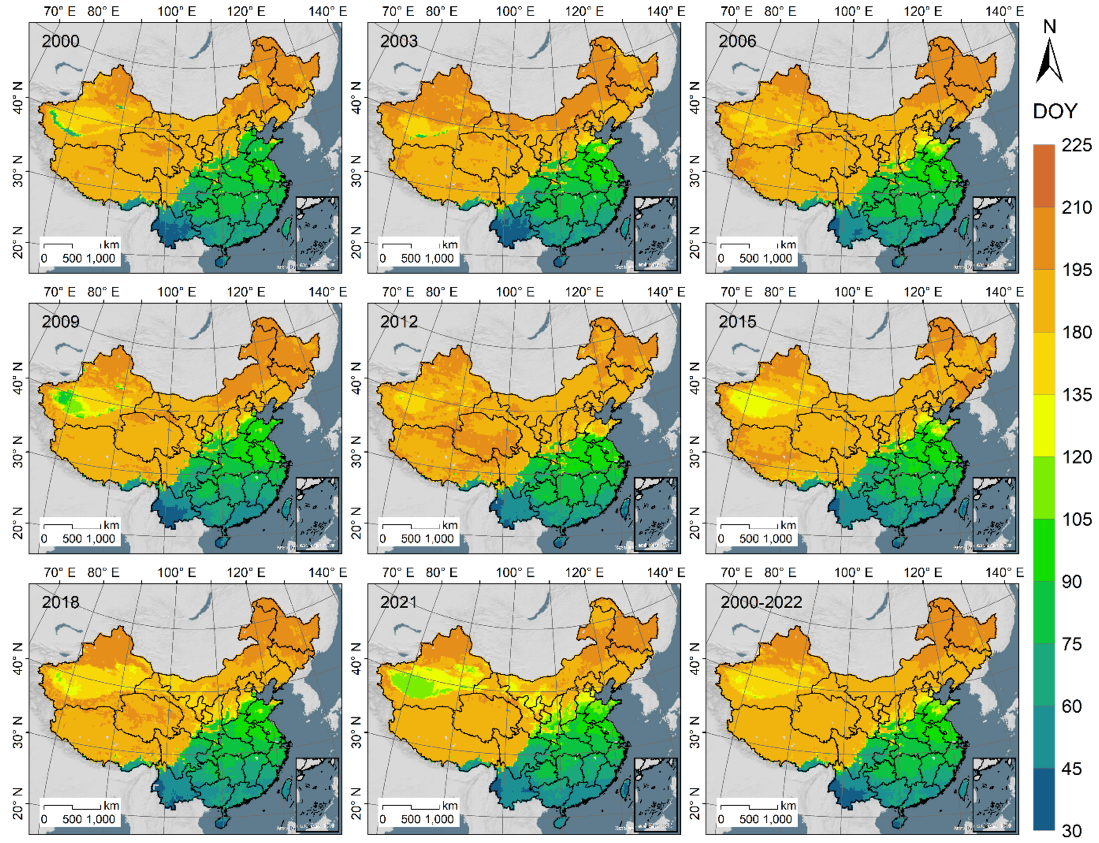

Fig.S2 Peak flowering date maps of rapeseed in China from 2000 to 2022.

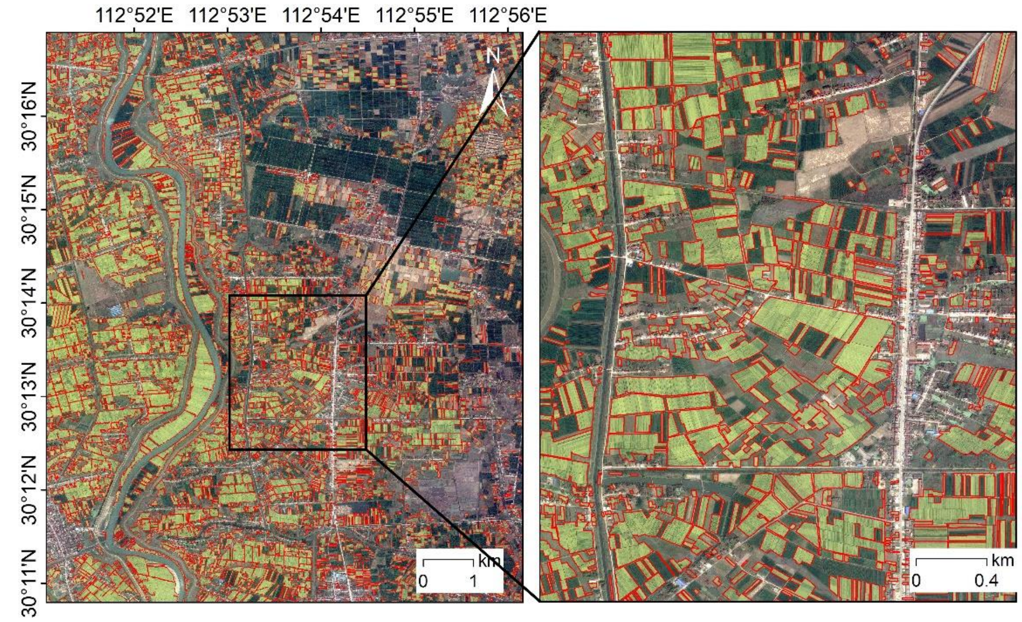

Fig.S3 Geographic location of the test area and labelled rapeseed vector polygons.
